# A simplified approach using Taqman low-density array for medulloblastoma subgrouping

**DOI:** 10.1186/s40478-019-0681-y

**Published:** 2019-03-04

**Authors:** Gustavo Alencastro Veiga Cruzeiro, Karina Bezerra Salomão, Carlos Alberto Oliveira de Biagi Jr, Martin Baumgartner, Dominik Sturm, Régia Caroline Peixoto Lira, Taciani de Almeida Magalhães, Mirella Baroni Milan, Vanessa da Silva Silveira, Fabiano Pinto Saggioro, Ricardo Santos de Oliveira, Paulo Henrique dos Santos Klinger, Ana Luiza Seidinger, José Andrés Yunes, Rosane Gomes de Paula Queiroz, Sueli Mieko Oba-Shinjo, Carlos Alberto Scrideli, Suely Marie Kazue Nagahashi, Luiz Gonzaga Tone, Elvis Terci Valera

**Affiliations:** 1 Department of Pediatrics Ribeirão Preto Medical School, Hospital das Clínicas, University of São Paulo, Av. Bandeirantes 3900, Ribeirão Preto, São Paulo Brazil; 2 Department of Genetics, Ribeirão Preto Medical School, University of São Paulo, Av. Bandeirantes 3900, Ribeirão Preto, São Paulo Brazil; 3 Department of Oncology, Children’s Research Center, Neuro-Oncology group, University Children’s Hospital Zürich, August-Forel Strasse 1, CH-8008 Zürich, Switzerland; 4 Pediatric Glioma Research Group, Hopp Children’s Cancer Center at the NCT Heidelberg (KiTZ) and German Cancer Research Center (DKFZ), 69120 Heidelberg, Germany; 5 Department of Pathology, University of São Paulo, Av. Bandeirantes 3900, Ribeirão Preto, SP 14049-900 Brazil; 6 Division of Pediatric Neurosurgery, Department of Surgery and Anatomy, Ribeirão Preto Medical School, Hospital das Clínicas, University of São Paulo, Av.Bandeirantes 3900, Ribeirão Preto, São Paulo Brazil; 7 Boldrini Centre of Children, University of Campinas-UNICAMP, Campinas, SP Brazil; 8 Department of Neurology, Faculty of Medicine, University of São Paulo, São Paulo, Brazil

**Keywords:** Medulloblastoma, Molecular subgroups, Brazilian cohort, Real-time PCR

## Abstract

Next-generation sequencing platforms are routinely used for molecular assignment due to their high impact for risk stratification and prognosis in medulloblastomas. Yet, low and middle-income countries still lack an accurate cost-effective platform to perform this allocation. TaqMan Low Density array (TLDA) assay was performed using a set of 20 genes in 92 medulloblastoma samples. The same methodology was assessed in silico using microarray data for 763 medulloblastoma samples from the GSE85217 study, which performed MB classification by a robust integrative method (Transcriptional, Methylation and cytogenetic profile). Furthermore, we validated in 11 MBs samples our proposed method by Methylation Array 450 K to assess methylation profile along with 390 MB samples (GSE109381) and copy number variations. TLDA with only 20 genes accurately assigned MB samples into WNT, SHH, Group 3 and Group 4 using Pearson distance with the average-linkage algorithm and showed concordance with molecular assignment provided by Methylation Array 450 k. Similarly, we tested this simplified set of gene signatures in 763 MB samples and we were able to recapitulate molecular assignment with an accuracy of 99.1% (SHH), 94.29% (WNT), 92.36% (Group 3) and 95.40% (Group 4), against 97.31, 97.14, 88.89 and 97.24% (respectively) with the Ward.D2 algorithm. t-SNE analysis revealed a high level of concordance (k = 4) with minor overlapping features between Group 3 and Group 4. Finally, we condensed the number of genes to 6 without significantly losing accuracy in classifying samples into SHH, WNT and non-SHH/non-WNT subgroups. Additionally, we found a relatively high frequency of WNT subgroup in our cohort, which requires further epidemiological studies. TLDA is a rapid, simple and cost-effective assay for classifying MB in low/middle income countries. A simplified method using six genes and restricting the final stratification into SHH, WNT and non-SHH/non-WNT appears to be a very interesting approach for rapid clinical decision-making.

**Electronic supplementary material:**

The online version of this article (10.1186/s40478-019-0681-y) contains supplementary material, which is available to authorized users.

## Introduction

Medulloblastoma (MB) is the most common malignant brain tumor of children and adolescents, representing 20% of all pediatric brain tumors and is considered to be a complex disease from a genetic perspective [31]. Current consensus divides MB into four main molecular subgroups: SHH, WNT, Group 3 and Group 4. These subgroups have distinct transcriptional profiles, copy-number aberrations, somatic mutations and clinical outcomes [2, 18, 22–24].

The molecular subgroups of MB have been incorporated into risk stratification along with conventional biomarkers and preclinical models to evaluate novel targeted inhibitors and to substantiate further clinical trials [21, 23, 31]. The updated World Health Organization (WHO) classification of the central nervous system (CNS) acknowledges some of these molecular features as risk-stratification factors for MB [17]. Still, low and even middle-income countries cannot afford to routinely use these next-generation sequencing (NSG) platforms for MB molecular subgrouping. High costs related to these new technologies (e.g Illumina Methylation array 450 k and NanoString nCounter®) preclude their routine clinical application in most low-income Nations.

As an initial attempt to equate this subject, Kunder and colleagues [15] described a miRNA-based real-time PCR assay platform that performed subgroup assignment using a reduced set of 21 probes. However, analyses of Group 3 and Group 4 MB subgroups were not precisely discriminative when this approach was used and no algorithm accuracy was validated for their method. Similarly, Kaur and colleagues [12] published a simplified approach based on immunohistochemistry (IHC) and real time PCR (qPCR) methods for MB subgroup allocation [12]. However, overlapping IHC staining was observed between subgroups. More recently, complete datasets from cohort studies have become publicly available, allowing the validation for new molecular classification and comparing novel stratification proposals for gold standard NGS data. Accordingly, the validation of new algorithms seems to be critical considering their increasing genomic and molecular importance for therapeutic decisions [4, 7, 10, 16].

Here, we describe a low-cost and straightforward method for molecular allocation of MB patients. We hypothesized that a combination of qPCR with precise algorithms would be a useful, simple and potent tool for molecular assignment of MB tumors. We have optimized the number of genes to molecularly classify patients into four and three groups of interest for clinical management. We also present an elucidative algorithm for MB subgroup assignment, validating our approach and comparing our findings to data from 763 MB samples molecularly assigned through a robust integrative methodology (transcriptional, methylation and cytogenetic profiles) (GSE85217), as well as confirming our subgroup findings by Methylation array in a sample subset.

## Methods

### Study group

**Fig. 1:**
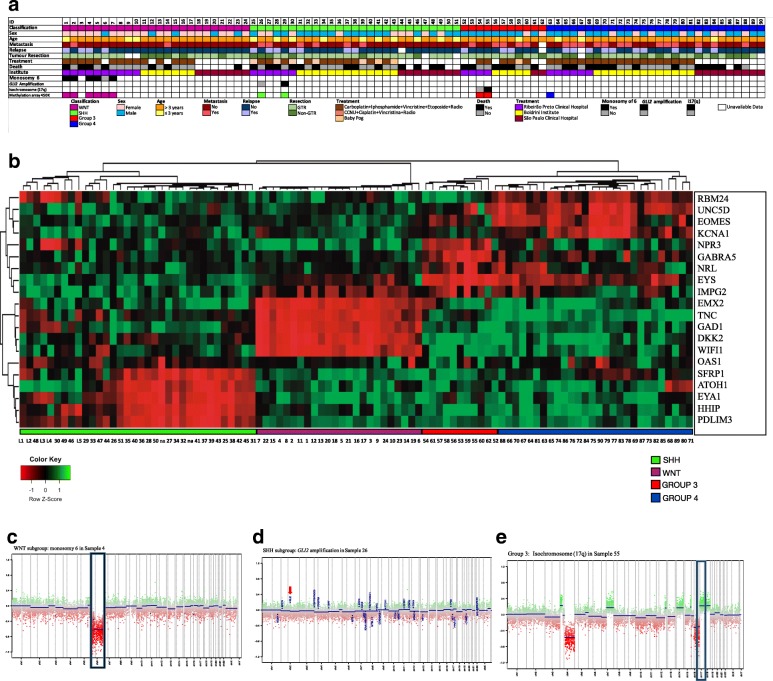
**a** Clinical characteristics of MB patients (*n* = 90). *Classification* Molecular classification: WNT subgroup, SHH subgroup, Group 3 and Group 4 of patients. *Gender* (female and male). *Age at diagnosis* (below or above 3 years). *Metastasis* presence of metastasis at diagnosis (yes, no); *Relapse* presence of postoperative disease relapse (yes, no). *Tumor resection* (gross-total resection GTR; non-gross total resection non-GTR). *Treatment* treatment protocol (craniospinal radiotherapy plus carboplatin, ifosfamide, vincristine, etoposide; craniospinal radiotherapy plus CCNU (1-(2-chloroethyl)-3-cyclohexyl-1-nitrosourea), cisplatin, vincristine; Baby POG – Pediatric Oncology Group). *Death* if patient died (yes, no). *Institution* institute where patients received treatment, *Monosomy of chromosome 6* if patient bears this feature (yes, no), *GLI2 Amplification* if patients bears feature (yes, no), Isochromosome (17q) if patient bears feature (yes, no), *Methylation Array 450 K* Molecular assignment by methylation array of WNT (6), SHH (2), Group 3 (2) and Group 4 (1) samples. **b** Hierarchical unsupervised clustering of 92 primary MB into four molecular subgroups: SHH (green), WNT (purple), Group 3 (red) and Group 4 (blue). Pearson distance as Metric and average linkage as algorithm clustering. L1, L2, L3, L4 and L5 are represented as UW473, DAOY, UW402, UW228 and ONS-76 MB cell lines and “na” as samples tumors with unavailable data. **c** Copy number profile of sample 4 WNT subgroup (monosomy 6) (d) Copy number profile of sample 26 SHH Subgroup (Amplification of *GLI2)* (e) Copy number profile of sample 55 Group 3 (Isochromosome 17q)

Ninety-two patients diagnosed with MB and treated at three Brazilian institutes were evaluated: 28 patients from the University Hospital, Ribeirão Preto Medical School, University of São Paulo (HC/FMRP-USP), 38 from The Boldrini Center of Children in Campinas São Paulo State, and 26 from the Medical School of São Paulo, University of São Paulo. In summary, 92 fresh-frozen MB tissue samples were microdissected by a single pathologist (F.S.P) in the Pathology Department (FMRP-USP). During microdissection, necrotic and normal tissues were removed from viable tumor areas. Patient age ranged from 1 to 24 years (median age = 7 years). Age groups at tumor diagnosis (clinical data from 88 MB patients out of 92 cases were available) were: infants (1–35 months) 11/88, children (36 months - 8 years) 38/88, and adolescents (9 to 17 years) 35/88. Tumors of young adults (age equals or above 18 + years) represented 4/88 of the case series. There was a slight male preponderance, with a male to female ratio of 1.30:1.0. From 92 samples, follow-up data of 80 patients were available and ranged from 1 to 168 months, with a median observation period of 41 months. Thirty-nine patients died because of the disease (DOD), 37 patients showed no evidence of disease (NED) at their last follow-up, and 4 patients (4.81%) died due to other unrelated events. Follow-up from a period of 1 to 5 years was available for 73% (59/80) of patients and 30% (24/80) were followed for more than 5 years. Two patients who lacked clinical information (named as “na” from HC-FMP/USP) were included in the heatmap for molecular assignment and were not considered for further analysis (clinical, demographic and survival (Additional file 1)). Patients’ clinical information, outcome, demographic and information on Methylation profile are presented in Fig. 1.

### TaqMan low density array (TLDA)

Microdissected fresh frozen tumor tissues were submitted to RNA extraction using the RNAeasy kit (Qiagen). cDNA was synthetized in duplicate in a 25 μl reaction volume using 500 ng RNA from the High Capacity Kit (Thermo). After RT-PCR, 25 μl of DEPC water and 50 μl of Universal Master Mix (Life Technologies) were added at a ratio of 1:1. The TLDA plate layout was 31 + 1. The plate layout manufacturing control used was *GAPDH* and *18S* and the reference genes used were *TBP*, *HPRT* and *GUS-B*. Genes used for molecular assignment was retrieved from Codeset described by Northcott and colleagues [18] and probe set and manufacturer’s code TaqMan probe are listed in (Additional file 2: Table S1). Four samples per plate were analyzed by conventional Real-Time PCR, with 1 μl Reaction Predesigned added to each well on Quant Studio 12 K flex (Thermo). The relative quantification (RQ) of each protein-coding gene compared with *TBP*, *HPRT* and *GUS-B* was determined by the comparative cycle threshold (Ct) method, where RQ 1/4 22(Ct Gene 2Ct Ref) × 100.

### Molecular assignment of MB samples

Codeset genes expression analysis was used to generate a pairwise distance matrix. Additionally, MB cell lines previously assigned to the SHH subgroup: DAOY, UW228, ONS-76 [9, 18, 29, 30] and the UW402 and UW473 (no subgroup information) were included in the analysis.

For molecular subgroup assignment, unsupervised hierarchical clustering was performed by Pearson distance correlation followed by an average-linkage algorithm. Delta Ct values were used during analysis and a Heatmap was generated using the Expression Suite® software (Life Technologies). A total of 763 MB samples from the study of Cavalli and colleagues [1] (GSE85217) in the R environment were analyzed for algorithm validation and heatmap comparison.

### Molecular assignment of MB samples by methylation array and copy number profiling

In order to assess concordance between TLDA assay and the gold standard Illumina 450 K Methylation array, DNA was extracted from 11 fresh frozen MB tumors and 250 ng were processed for genome-wide DNA methylation analysis using the Illumina HumanMethylation450 BeadChip (450 k). t-SNE analysis (t-Distributed Stochastic Neighbor Embedding, Rtsne package version 0.11) was performed and MB samples were randomly tested along with 390 MB reference samples molecularly assigned in Capper and colleagues study (GSE109381) [1]. MB samples were further submitted to DNA methylation class prediction and calibrated random forest class prediction scores were generated using classifier version 11. b4 based on the analysis of 10,000 CpG sites present in the 450 k. For molecular subgrouping based on methylation class, an optimal calibrated score threshold was defined as ≥0.5 for a sufficient prediction as long as all family member scores add up to a total score of ≥0.9. Additionally, copy-number variations (CNV) analysis was performed using the ‘conumee’ R package in bioconductor. Briefly, all CpGs are represented by a methylation probe and unmethylated probe. For CNVs identification, the methylated and unmethylated signal intensities are added together and a ratio is formed against a healthy reference (e.g normal cerebellum tissue) that bears a flat genome. Finally, the relative copy-number is plotted in the corresponding area of chromosomal location. The automatic score is verified by manual curation of the respective loci for each individual profile [8, 29].

### Bioinformatic analysis

The R language and environment for statistical computing and graphics was used for bioinformatic analysis. The ComplexHeatmap and circlize packages were used for Heatmap generation [5, 6] and the ggplot2package [26, 32] was used for graphics generation. Rtsne [14, 10] was used for the visualization of t-Distributed Stochastic Neighbor Embedding (t-SNE) and the NbClust and Factoextra packages [3, 11] were used to point out the best number of clusters and to visualize the results. Pearson correlation was used as a distance parameter and we selected the clustering algorithms Ward.D2 or Average linkage. To perform t-SNE, a method for constructing a low dimensional embedding of high-dimensional data, distances or similarities, we used the default parameters, setting only the perplexity parameter at 30, with 5–50 being the typical and recommended range for robust analysis. We then used NbClust with default parameters to find the best number of cluster and to visualize the results.

### Statistical analysis

The SPSS version 20 software (SPSS Inc., Chicago, IL, USA) was used for statistical analysis. Descriptive statistics and frequency distributions were calculated for all variables; the chi-square test and Fisher’s exact test were applied to explore association between variables. Patients’ event-free survival (EFS) rates were evaluated by Kaplan-Meier curves and the log-rank test, considering relapse/death as the event. *P*-values < 0.05 were considered to be significant. [* < 0.05, ** < 0.01, *** < 0.001].

## Results

### TLDA accurately assigned most of the MB samples as WNT, SHH, Group 3 and Group 4

As previously described [13, 15, 19] the SHH MB subgroup was defined according to the expression of *PDLIM3*, *EYA1*, *HHIP*, *ATOH1*, *SFRP1,* with *EYA1 HHIP* and *SFRP1* being the genes most expressed in 27 SHH MB patients (30%). The WNT subgroup in 24 patients (27%) was represented by the *OAS1*, *WIFI1*, *DKK2*, *TNC*, *GAD1* and *EMX2* genes, with *WIFI1*, *DKK2* and *EMX2* being the most expressed genes in this subgroup. *IMPG2*, *EYS*, *NRL*, *GABRA5* expression was used to assign 11 MB samples (12%) to Group 3, and *GABRA5* and *NPR3* expression was the most specific subgroup compared to Group 4. Twenty-eight Group 4 MBs (31%) were assigned using *KCNA1*, *EOMES*, *UNC5D*, and *RBM24* expression, with *KCNA1* and *RBM24* being the most specific subgroup compared to Group 3. Specifically, *TNC* showed higher expression in WNT subgroup and average expression in SHH MB. The *EYS* gene was differentially expressed in Group 3 and Group 4 (Fig. 1b). DAOY, UW228 and ONS-76 cell lines were confirmed to belong to SHH subgroup. UW473 and UW402 were subgrouped as SHH MB as well.

### Methylation and copy number profiling of MBs using illumina methylation array 450 K showed high concordance with TLDA

In order to validate our method, DNA available of 11 randomized MB patients were submitted to Methylation array 450 K (Copy number profile available in Fig. 1). We found a high concordance between Methylation array 450 K and TLDA for molecular assignment of MBs. The t-SNE analysis of eleven MB samples along with 390 MB samples (GSE109381) showed high concordance with TLDA method, being all samples assigned in the same molecular subgroup (Additional file 3: Figure S1). The DNA methylation class prediction and calibrated random forest class prediction scores identified 6 WNT MBs, 2 SHH MBs, two Group 3 MBs and one Group 4 MB (Additional file 4: Table S2). Additionally, copy number profiling identified monosomy in chromosome 6 in WNT subgroup (*n* = 5), *GLI2* amplification in SHH (*n* = 1) and I (17q) for Group 3 MBs (*n* = 1) (Fig. 1c, d and e respectively).

### T-SNE analysis revealed concordance between the Brazilian cohort and the validation cohort and highlighted overlapping features of group 3 and group 4

**Fig. 2:**
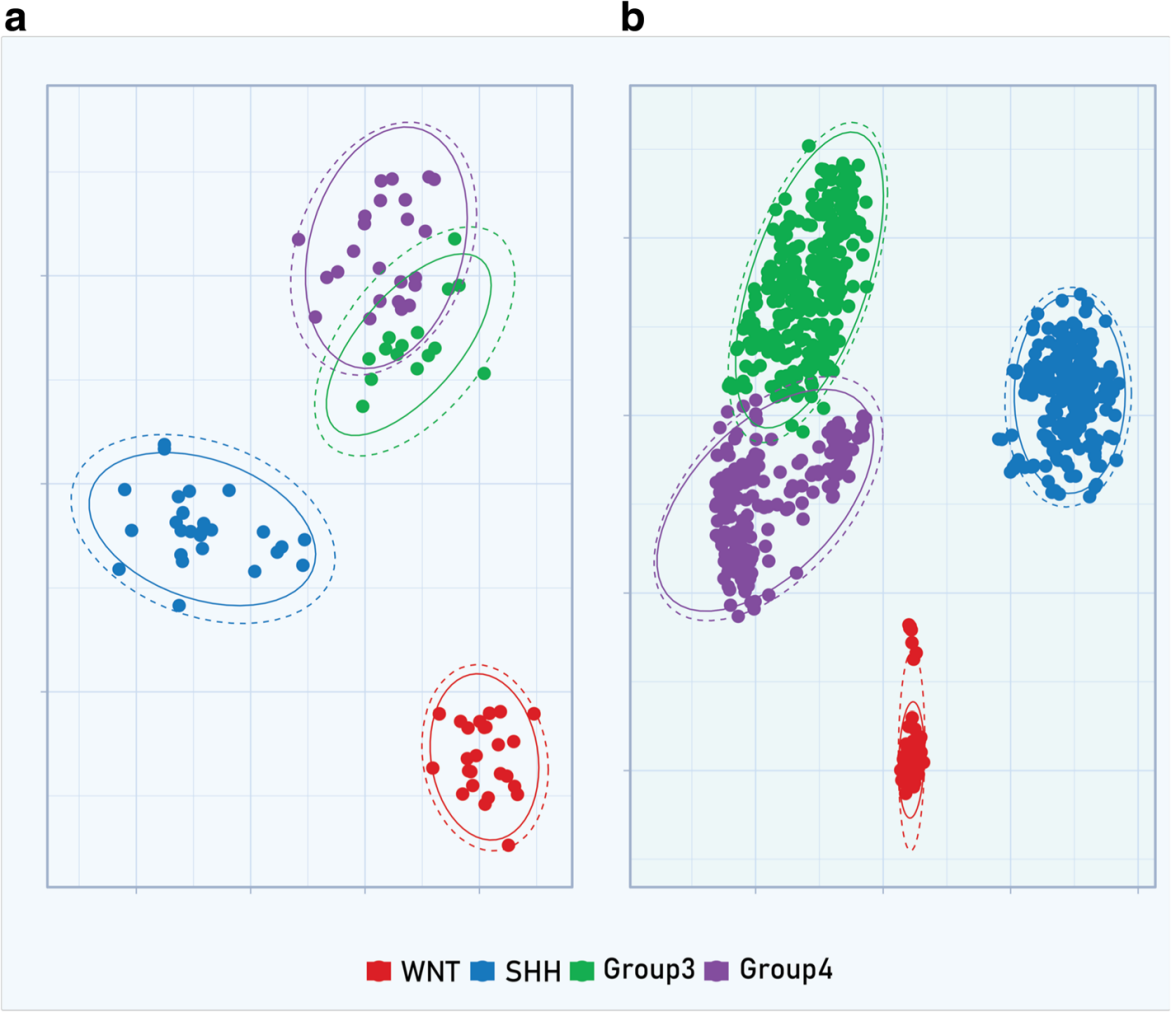
**a** Two-dimensional representation of pairwise sample correlations of twenty TaqMan expression assay probes (Additional file: Table S1) in 92 MB Brazilian samples by t-Distributed Stochastic Neighbor Embedding. **b** Two-dimensional representation of pairwise sample correlation of the same gene set represented in (**a**) using Microarray probes in 763 MB samples from GSE85217 by t-Distributed Stochastic Neighbor Embedding

t-SNE analysis was performed to visualize clustering features of molecular subgroups in perplexity index of 30. We found four subgroups in the Brazilian cohort study, with Group 3 and Group 4 bearing overlapping features (*k* = 4). To validate this analysis, the t-SNE algorithm was also applied to the validation cohort of 763 MB samples and the data obtained showed the same behavior (*k* = 4) (Fig. 2a and b).

### Average linkage and Ward.D2 are robust algorithms for subgroup assignment of MB

**Fig. 3:**
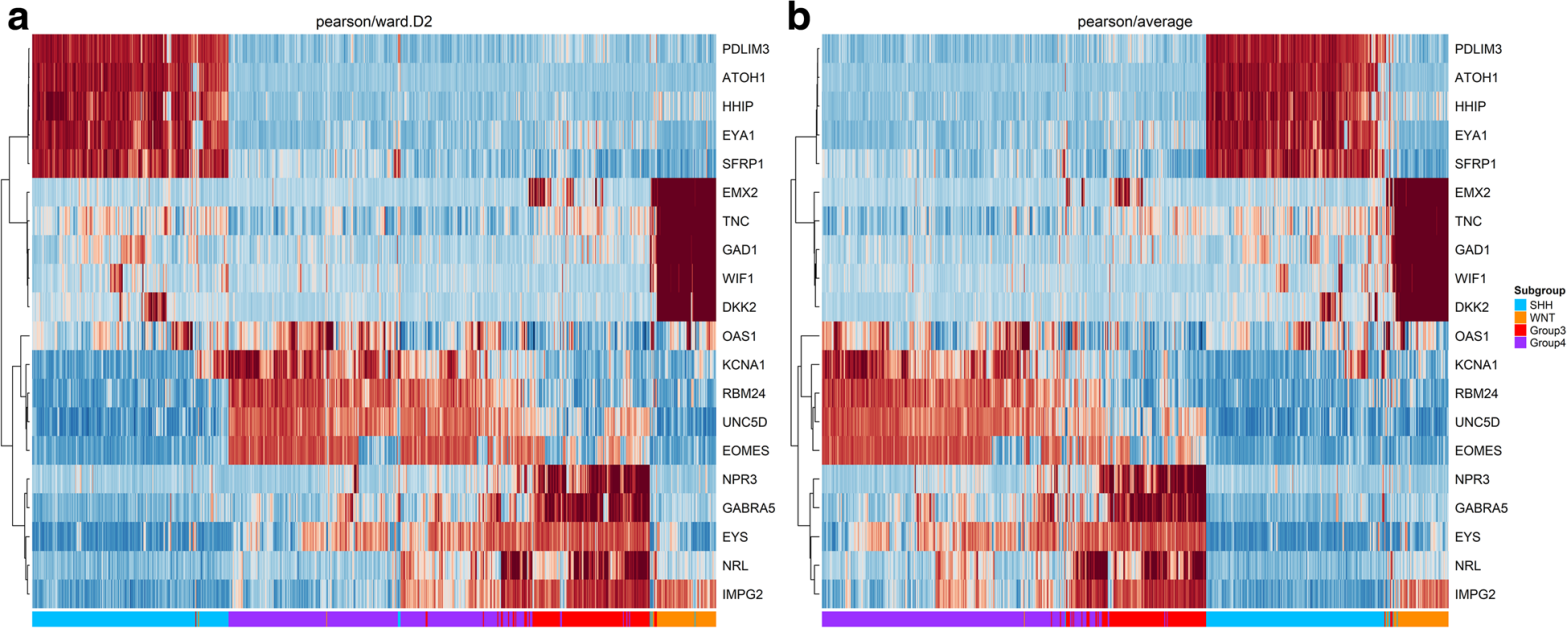
Hierarchical unsupervised clustering of previously classified 763 primary MB in GSE85217 study: SHH (blue), WNT (orange), Group 3 (red) and Group 4 (purple) Pearson distance as Metric was utilized in both heatmaps. **a** Clustering using the Ward.D2 algorithm. **b** Clustering using the Average linkage algorithm

In order to compare the clusterization feature algorithms Ward and Average-linkage we applied our TLDA approach to a validation cohort of 763 pre-classified MB samples submitted to an integrative methodology composed of transcriptional, methylation profile and cytogenetic features. Interestingly, we found both Average-linkage and Ward.D2 to be feasible algorithms for MB subgroup assignment using transcriptional data alone. The Average-linkage algorithm successfully assigned 221 of 223 SHH MB samples (99.10% accuracy), 66 from 70 WNT MB samples (94.29% of accuracy), 133 from 144 MB Group 3 MB samples (92.36% accuracy), and 311 from 326 Group 4 MB samples (95.40% accuracy). Equally, the Ward.D2 algorithm successfully assigned 216 of 223 SHH MB samples (97.31% accuracy), 68 from 70 WNT MB samples (97.14% accuracy), 128 from 144 MB Group 3 MB samples (88.89% accuracy), and 317 from 326 Group 4 MB samples (97.24% accuracy). (Fig. 3a and b) (Table 1).

**Table 1:**
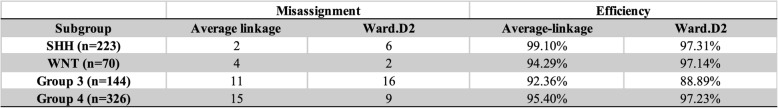
Comparison of algorithm accuracy in the GSE85217 study (*n* = 763). Misassignment is defined as patients who were incorrectly subgrouped

### Analysis of *SFRP1*, *HHIP*, *EYA1*, *WIFI1*, *EMX2* and *DKK2* expression potentially discriminated between SHH, WNT from non-SHH/non-WNT MB

**Fig. 4:**
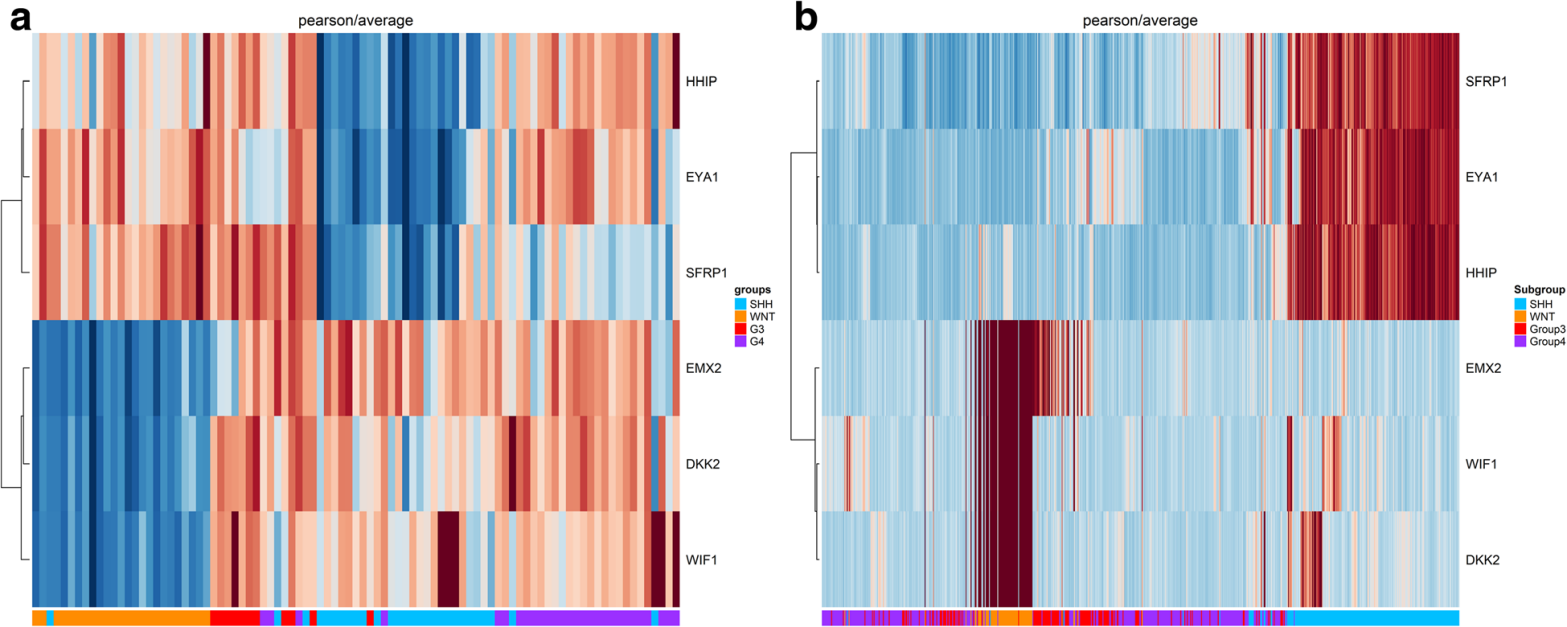
Hierarchical unsupervised clustering using *HHIP*, *EYA1*, *SFRP1*, *EMX2*, *DKK2*, *WIFI1* **a** 92 MB samples from Brazilian cohort and **b** 763 MB samples from GSE85217. SHH (blue), WNT (orange), Group 3 (red) and Group 4 (purple)

**Fig. 5:**
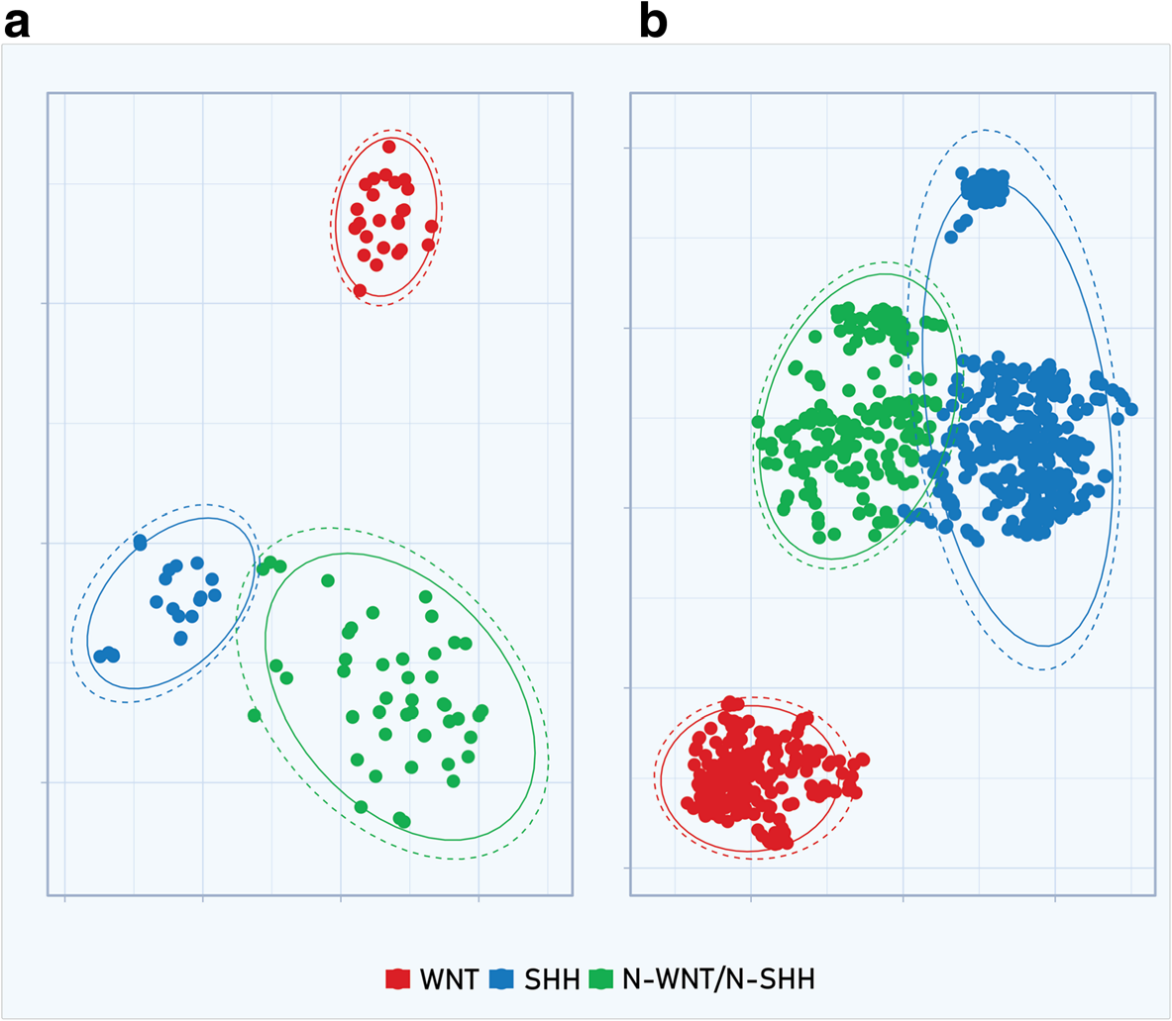
**a** Two-dimensional representation of pairwise sample correlations of 6 TaqMan expression assay probes (*SFRP1*, *HHIP*, *EYA1*, *WIFI1*, *EMX2* and *DKK2)* in 92 MB Brazilian samples by t-Distributed Stochastic Neighbor Embedding. **b** Two- dimensional representation of pairwise sample correlation of the same gene set represented in (**a**), although using Microarray probes of 763 MB samples from GSE85217 by t-Distributed Stochastic Neighbor Embedding

We further performed expression analysis of six key genes that bear a positive signature for SHH (*SFRP1*, *HHIP*, *EYA1*) and WNT (*WIFI1*, *EMX2* and *DKK2*) using Pearson as distance measurement and Ward.D2 or Average linkage as clustering algorithms. We found that the first cluster was characterized by a differential expression of *SFRP1*, *HHIP* and *EYA1*, which represent the SHH subgroup. Another cluster that differentially carried expression of *WIFI1*, *EMX2* and *DKK2* represented the WNT subgroup. The third cluster, which carried very low levels or lacked expression of the six genes, was assigned as N-WNT/N-SHH. Similarly, in the validation cohort of 763 samples, we identified the same behavior, indicating the presence of three main clusters (Fig. 4a and b). Using t-SNE analysis, we observed the same consistent assignment of MB samples to 3 main clusters (*k* = 3), with a minor overlap of clusters N-SHH/N-WNT and SHH (Fig. 5a and b) (Additional file 5: Figure S2a and b). The accuracy of subgroup assignment using the set of six genes is showed in Table 2a and b.

**Table 2:**
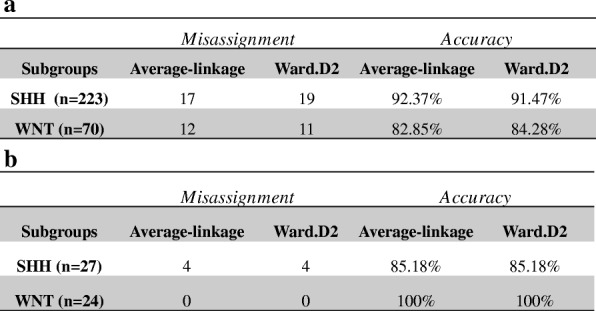
Comparison of algorithm accuracy using 6 genes to assign WNT and SHH alone. (**a**) Study GSE85217 (*n* = 293). (**b**) Brazilian cohort (*n* = 51). Misassignment is defined as patients who were incorrectly subgrouped

## Discussion

In the present study, differential expression analysis of 20 genes from the CodeSet described by Northcott and colleagues [19] by TDLA approach permitted us to molecularly assign a cohort of 92 MB patients to the four major MB subgroups. Additionally, we validated the same gene set in a cohort of 763 MB patients from the GSE85217 reference study, which applied the integrative-clustering method to molecularly classify MB samples. The WNT and SHH subgroups were robustly identified since they formed a solid and concise cluster generated by the Average-linkage or Ward.D2 algorithms and confirmed by t-SNE analysis. In agreement, similar patterns were detected using GSE85217 data analysis. We demonstrated that assessment of the transcription profile is not sufficient to completely discriminate all Group 3 MB from Group 4 MB since a minority of these patients share transcription and common molecular features [10, 12, 15, 18].

Next, in order to exam the concordance of our TDLA approach with NGS subgrouping for MB we validated molecular assignment of 11 MBs samples by Methylation Array 450 K. We found a high frequency of monosomy in chromosome 6 within WNT (5 out of 6) subgroup corroborating with previous studies [2, 8, 13, 28]. In one SHH MB samples evaluated by Methylation array we identified *GLI2* amplification. For Group 3, one MB specimen bears isochromosome 17q, a reliable marker for this subgroup [28] (Fig. 2B). Only one sample for group 4 was identified, and it also clustered to group 4 by TLDA method accordingly. Full concordance between eleven MB samples by NGS and TDLA was observed. Despite only a small set of samples was assessed, the results from NGS data support our molecular assignment provided by TLDA [2, 8, 13, 28].

In the present study, we found 27% of WNT MBs (Additional file 6: Figure S3a-S3d and Additional file 7: Figure S4). Although this is a high frequency when compared to studies performed in North American and European continents [19], Kunder and colleagues [15] reported 24% of WNT MBs in an Indian cohort. Moreover, pediatric neoplasms subtypes vary in frequency depending on the genetic population background (i.e: high frequency of Promyelocitic Leukemia in Latin America) [20, 25]. Interestingly, we found 2 cases of desmoplastic and 1 LCA in WNT MBs. Besides it is unlikely to find desmosplastic histological variants in WNT MBs, our data are supported by other studies [27]. In summary, these epidemiological facts highlight the urge for a reliable, feasible and low-cost method to perform molecular assignment of MBs in low and middle-income countries.

The average-linkage and Ward.D2 algorithms were assessed regarding their clustering features and subgroup assignment. In the GSE85217 study conducted on 763 MB patients, average-linkage provided better accuracy for SHH and Group 3 assignment compared to the Ward.D2 method. However, Ward.D2 was able to accurately classify WNT and Group 4 tumors. Interestingly, the pick of an accurate clustering algorithm may be subgroup specific. However, it is very important to understand the limitations of transcriptional data and information that can be extracted from a single feature such as gene expression [2, 27, 33].

Indeed, as reported by Cavalli and colleagues [2], the gold standard method for subgroup assignment is the assessment of the molecular features of the patient (transcription profile, methylation profile, cytogenetic profile) along with clinical information. However, in low-income countries, most molecular techniques are onerous for application to daily clinical practice. Using expression analysis of a gene set, algorithm assessment and bioinformatic analysis, we sought to identify the minimal number of genes needed to molecularly classify MB as WNT, SHH and non-SHH /non-WNT. In our study, by using a set of six differentially expressed genes we were able to distinguish SHH and WNT from non-WNT/non-SHH without s significant loss of accuracy. Both the Average-linkage and Ward.D2 algorithms conserved 100% accuracy for assignment to the WNT subgroup, with a decline to 85.18% for the SHH subgroup. As shown in the t-SNE map, there was a minor overlapping of samples of the non-SHH/non-WNT cluster with those of the SHH cluster. Additionally, we found high concordance between our data set and GSE85217, with 100% accuracy for the WNT subgroup and 86% accuracy for SHH. These results shed new light on a potential method for low-income countries based on a simple and feasible technique such as qPCR along with six probe/primer pairs plus reference genes with implementation of an approach recently described by Gómes and colleagues [3]. Their method fully discriminates between Group 3 and Group 4 based on the methylation status of 5 CpG’s, which is feasible for the real-time PCR platform through High Resolution Melting technology, and shall improve the molecular assignment [26].

Northcott et al. described a molecular classification method for MB that relies on the NanoString nCounter System. Besides the high accuracy of the method (~ 98%), the average cost is estimated at 60.00 USD per sample and the method takes 3–4 days to perform bioinformatic analysis [19]. The same method was reproduced by Leal and colleagues [16]; however, due to the high cost of the equipment (287,817.60 USD – average price in South America; 2018), it is challenging for most low-income countries to apply this method to clinical routine. Kaur et al. proposed a minimal panel comprising a combination of IHC antibodies and FISH probes to classify MB, with an estimate cost around 150.00 to 250.00 USD (average) per sample [12]. Although feasible, their approach does not seem to be as cost-effective as other methods and IHC analysis remains challenging due to different antibody batches and inter-observer consistency [19]. More recently, the minimal methylation classifier (MIMIC) was described as a highly efficient methodology that might be superior to Illumina 450 K and Methylation EPIC array for MB molecular assignment regarding feasibility for clinical routines; however, the average cost per sample with this approach is around 200.00 USD [26] and requires the acquisition of a MALDI-TOF mass-spectrometer (approximately 150,000.00 USD), along with a conventional PCR device. Our method using TLDA has an estimated cost of 70.00 USD per sample (including reagents, primers and laboratory implements). The equipment necessary to run TLDA costs about 92,600.00 USD and complete data analysis is ready within one working day. Moreover, when we condensed the number of studied genes to six (a set of TaqMan probes for: *SFRP1*, *HHIP*, *EYA1*, *WIFI1*, *EMX2* and *DKK2* along with reference genes *HPRT* and *Gus-ß*), the cost of molecular assignment to the WNT, SHH and N-SHH/N-WNT MB subgroups dropped to 26.82 USD per sample. Also, the real time-PCR (30,000.00 USD) platform is relatively inexpensive and commonly available in most hospitals due to its ample use for other routine laboratory applications. Finally, another advantage of the qPCR method is that it does not require batched minimal number of samples per run, being readily available to run single tumor samples upon arrival at the laboratory.

## Conclusions

In conclusion, we have developed a simplified approach and validated TDLA method in random samples by Methylation Array 450 K. In addition, our findings were challenged at a large cohort study GSE85217 through accurate algorithms for molecular assignment of MB. The proposed assay is cost-effective and discriminates most of SHH, WNT, non-SHH/non-WNT tumors. The TLDA method for MB subgroup stratification might be an affordable tool to be used to drive therapies in low-income countries. Moreover, it may also be an important approach for prompt classification and decision-making algorithms in MB, before NGS data analysis become available.

## Additional files

Additional file 1: Demographic analysis from our cohort study. Corresponding figures are Additional file 6: Figure S3a, S3b, S3c and S3d. (DOCX 13 kb)

Additional file 2: **Table S1.** TaqMan probes from the gene set used for MB molecular assignment. (CSV 787 bytes)

Additional file 3: **Figure S1.** t-SNE map show molecular assignment by Methylation array 450 K of 11 MB samples from our study along with 390 MB samples from GSE109381. (PDF 61 kb)

Additional file 4: **Table S2.** Report of DNA class prediction classifier using Random forest class prediction scores (classifier version 11. b4). (CSV 564 bytes)

Additional file 5: **Figure S2.** Comparison of clustering algorithms in our study (*n* = 92) with 6 genes *HHIP*, *EYA1*, *SFRP1*, *EMX2*, *DKK2*, *WIFI1.* (**a**) Ward.D2 algorithms (**b**) Average-linkage algorithms. (PDF 1980 kb)

Additional file 6: **Figure S3.** (**a**) Demographic distribution of the 4 molecular subgroups in the present cohort; (**b**) subgroup distribution with respect to age at diagnosis; (**c**) gender; (**d**) histological variants. The numbers indicate the sum of tumors in each category. (PDF 85 kb)

Additional file 7: **Figure S4.** Overall survival of molecular subgroups (*n* = 80). (PDF 62 kb)
