# Supplementary material for: A simplified approach using Taqman low-density array for medulloblastoma subgrouping
Source: Acta Neuropathol Commun. 2019 Mar 4;7:33. doi: 10.1186/s40478-019-0681-y (PMC6398239; doi:10.1186/s40478-019-0681-y)
Supplement: Supplementary file 1 — Demographic analysis from our cohort study. Corresponding figures are Additional file 6: Figure S3a, S3b, S3c and S3d. (DOCX 13 kb) [file 40478_2019_681_MOESM1_ESM.docx]

**Additional files #1**

**1) Demographic analysis revealed a high prevalence of the WNT subgroup in the Brazilian cohort.**

Demographic analysis of the Brazilian cohort (90 samples out of 92 patients) identified 27 cases (30%) of SHH, 28 Group 4 (31%), 24 WNT (27%) and 11 Group 3 (12%) (**Fig. S3a**). Tumors in children ≤ 3 years of age were predominantly SHH (80%), Group 3 (10%) and Group 4 (10%). In children between 3-8 years we observed a higher incidence of Group 4 (44%), followed by SHH (21%), WNT (18%) and Group 3 (11%). In older children (9-17 years) we observed WNT (34%), Group 4 (28%), SHH (25%) and Group 3 (11%) (**Fig. S3b**). In young adults we identified 3 WNT cases (75%) and 1 SHH (25%). Finally, for gender distribution we found a high male preponderance in SHH (1.45 : 1.00), Group 3 (4.5 : 1.00) and Group 4 (2.11 : 1.00) subgroups, while female gender was more frequent in WNT subgroup (0.26 : 1.00) (**Fig. S3c)**.

Most MB tumors showed classic histology (54%), followed by desmoplastic (31%), large cell anaplastic (LCA) (9.8%) and with extensive nodularity (MBEN) (5.2%). The SHH subgroup predominantly showed desmoplastic and LCA histology exclusively in MBEN tumors, while the WNT subgroup showed classic and desmoplastic histology. Samples belonging to Group 3 and Group 4 were identified predominantly in Classic and LCA MB, respectively (**Fig. S3d**).

**2) Survival analysis of molecular subgroups**

Overall survival data were available for 80 MB patients and Kaplan–Meier analysis showed the best survival rate for the WNT subgroup of patients, followed by SHH and Group 3, with the worst survival rate for Group 4 (**Fig. S4**). The log-rank test showed distinct overall survival (OS) curves when comparing WNT (74% ± 18%) to SHH (44.5% ± 12.2%; p=0.056), Group 3 (40% ± 15.5%; p=0.033) and Group 4 (35.7% ± 13.4%; p=0.057).
