# Supplementary material for: A simplified approach using Taqman low-density array for medulloblastoma subgrouping
Source: Acta Neuropathol Commun. 2019 Mar 4;7:33. doi: 10.1186/s40478-019-0681-y (PMC6398239; doi:10.1186/s40478-019-0681-y)
Supplement: Supplementary file 3 — Figure S1. t-SNE map show molecular assignment by Methylation array 450 K of 11 MB samples from our study along with 390 MB samples from GSE109381. (PDF 61 kb) [file 40478_2019_681_MOESM3_ESM.pdf]

S1

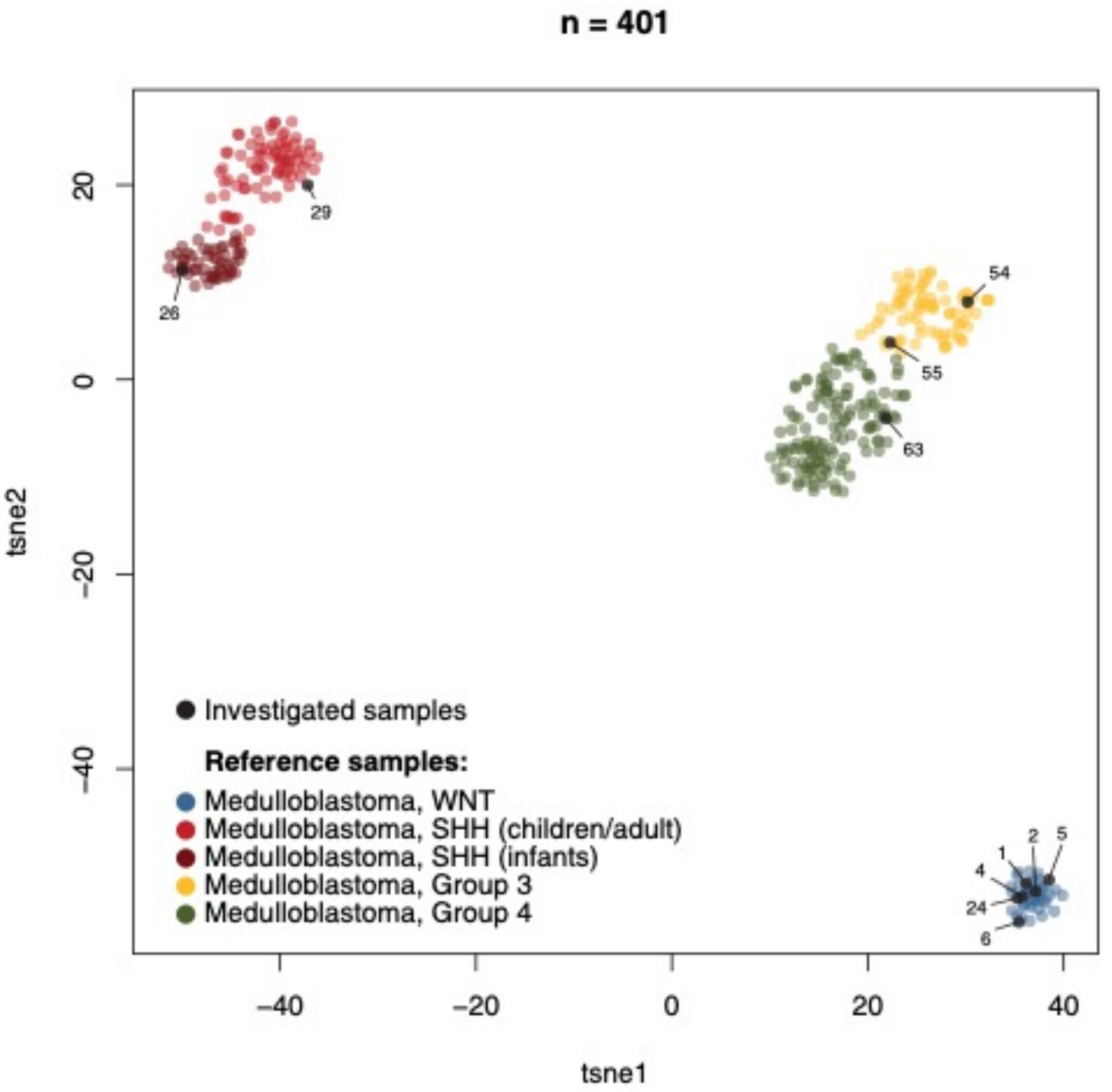

Fig. S1 t-SNE map show molecular assignment by Methylation array 450K of 11 MB samples from our study along with 390 MB samples from GSE109381.
