# Supplementary material for: A simplified approach using Taqman low-density array for medulloblastoma subgrouping
Source: Acta Neuropathol Commun. 2019 Mar 4;7:33. doi: 10.1186/s40478-019-0681-y (PMC6398239; doi:10.1186/s40478-019-0681-y)
Supplement: Supplementary file 5 — Figure S2. Comparison of clustering algorithms in our study (n = 92) with 6 genes HHIP, EYA1, SFRP1, EMX2, DKK2, WIFI1. (a) Ward.D2 algorithms (b) Average-linkage algorithms. (PDF 1980 kb) [file 40478_2019_681_MOESM5_ESM.pdf]

S2a

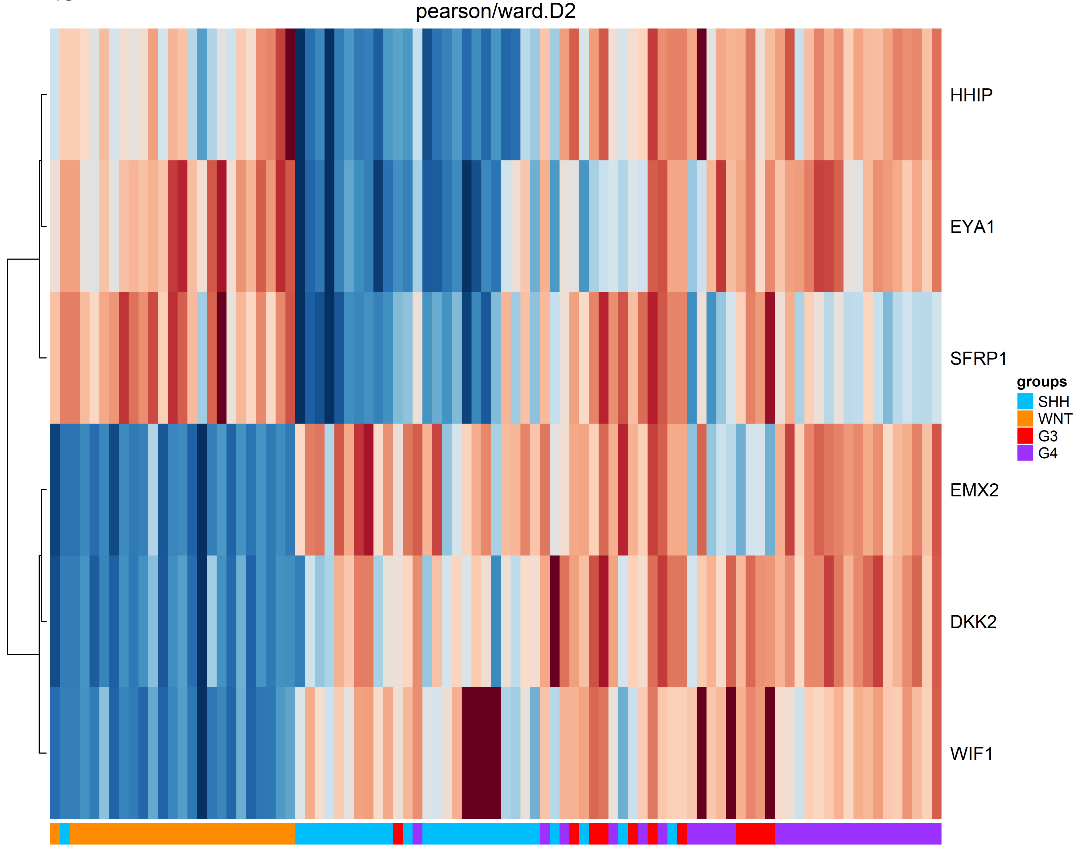

S2b

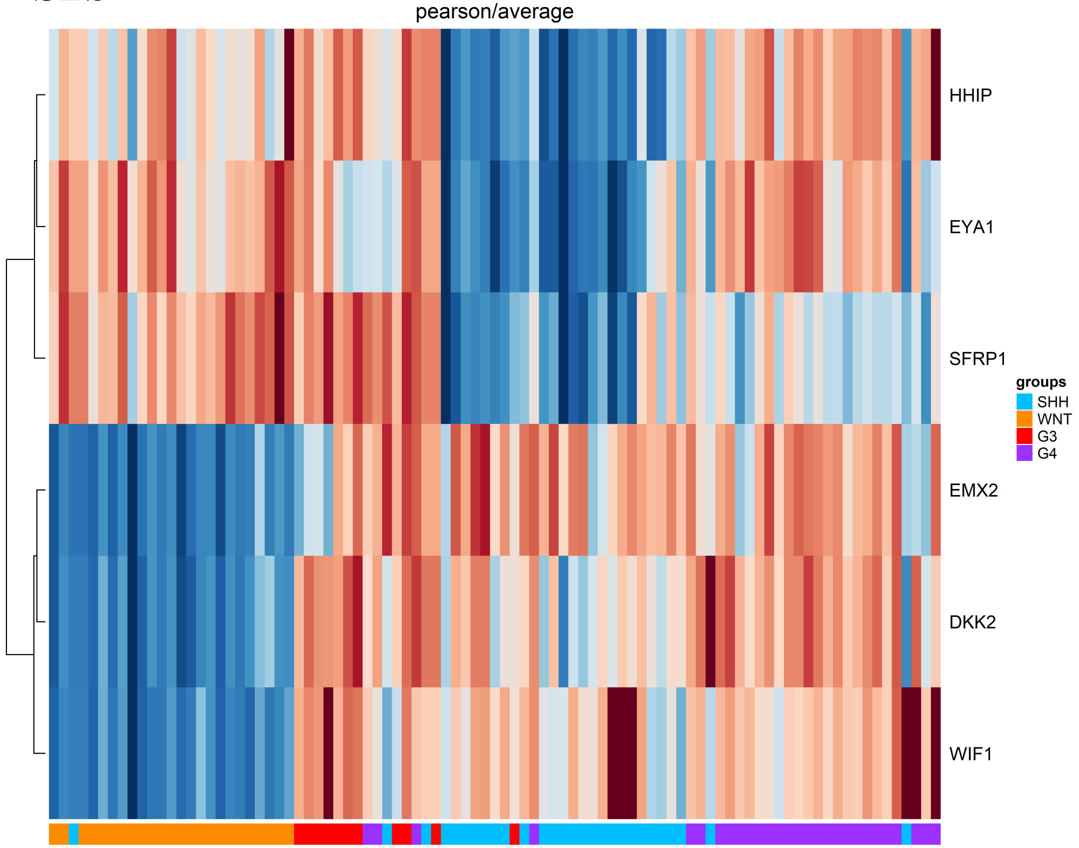

Fig. S2. Comparison of clustering algorithms in our study (n=92) with 6 genes *HHIP*, *EYA1*, *SFRP1*, *EMX2*, *DKK2*, *WIFI1*. **(a)** Ward.D2 algorithms **(b)** Average-linkage algorithms.
