# Supplementary material for: A simplified approach using Taqman low-density array for medulloblastoma subgrouping
Source: Acta Neuropathol Commun. 2019 Mar 4;7:33. doi: 10.1186/s40478-019-0681-y (PMC6398239; doi:10.1186/s40478-019-0681-y)
Supplement: Supplementary file 6 — Figure S3. (a) Demographic distribution of the 4 molecular subgroups in the present cohort; (b) subgroup distribution with respect to age at diagnosis; (c) gender; (d) histological variants. The numbers indicate the sum of tumors in each category. (PDF 85 kb) [file 40478_2019_681_MOESM6_ESM.pdf]

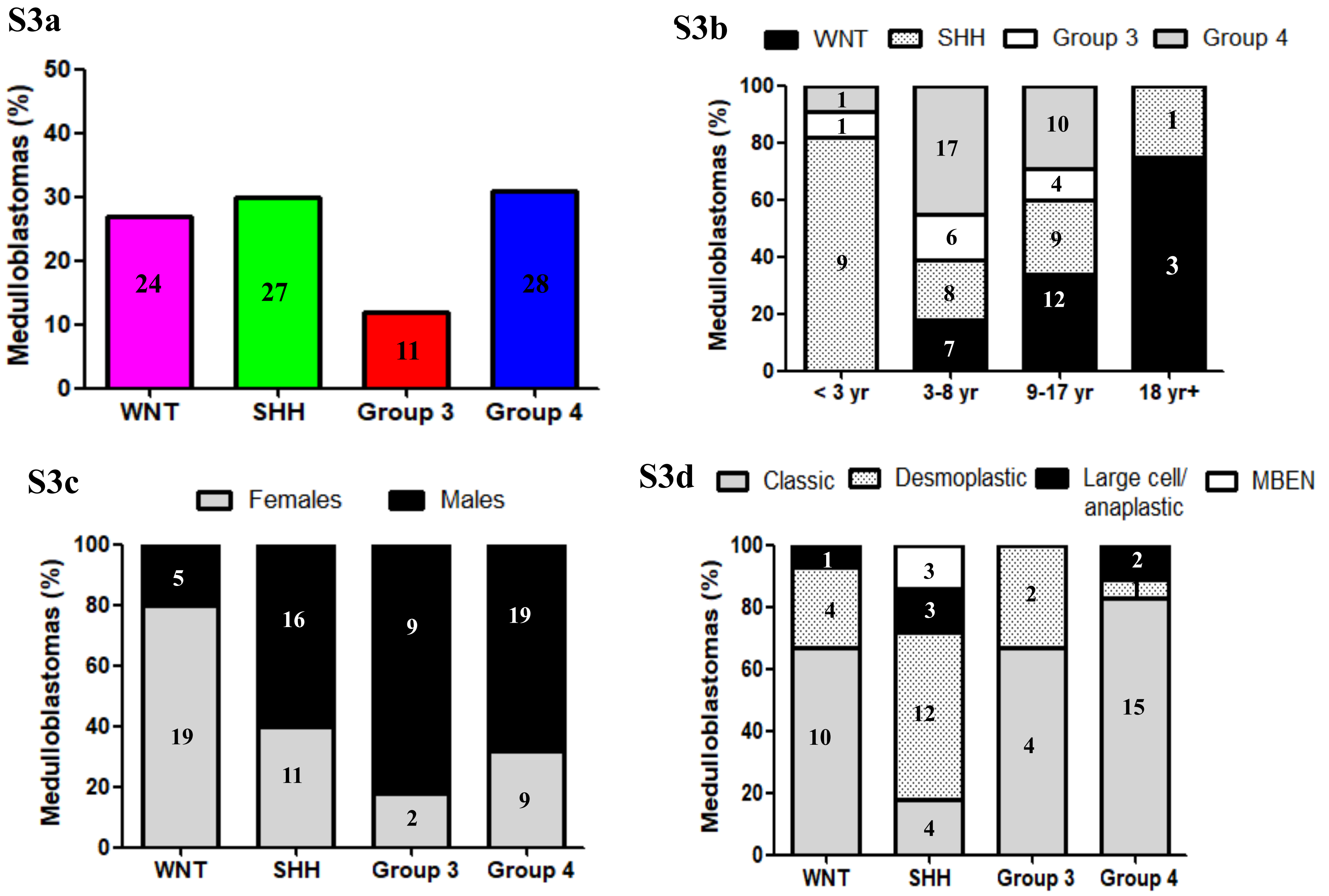

Fig. S3 **(a)** Demographic distribution of the 4 molecular subgroups in the present cohort; **(b)** subgroup distribution with respect to age at diagnosis; **(c)** gender; **(d)** histological variants. The numbers indicate the sum of tumors in each category.
