# Supplementary figures and images for: A simplified approach using Taqman low-density array for medulloblastoma subgrouping
Source: Acta Neuropathol Commun. 2019 Mar 4;7:33. doi: 10.1186/s40478-019-0681-y (PMC6398239; doi:10.1186/s40478-019-0681-y)

**S4**

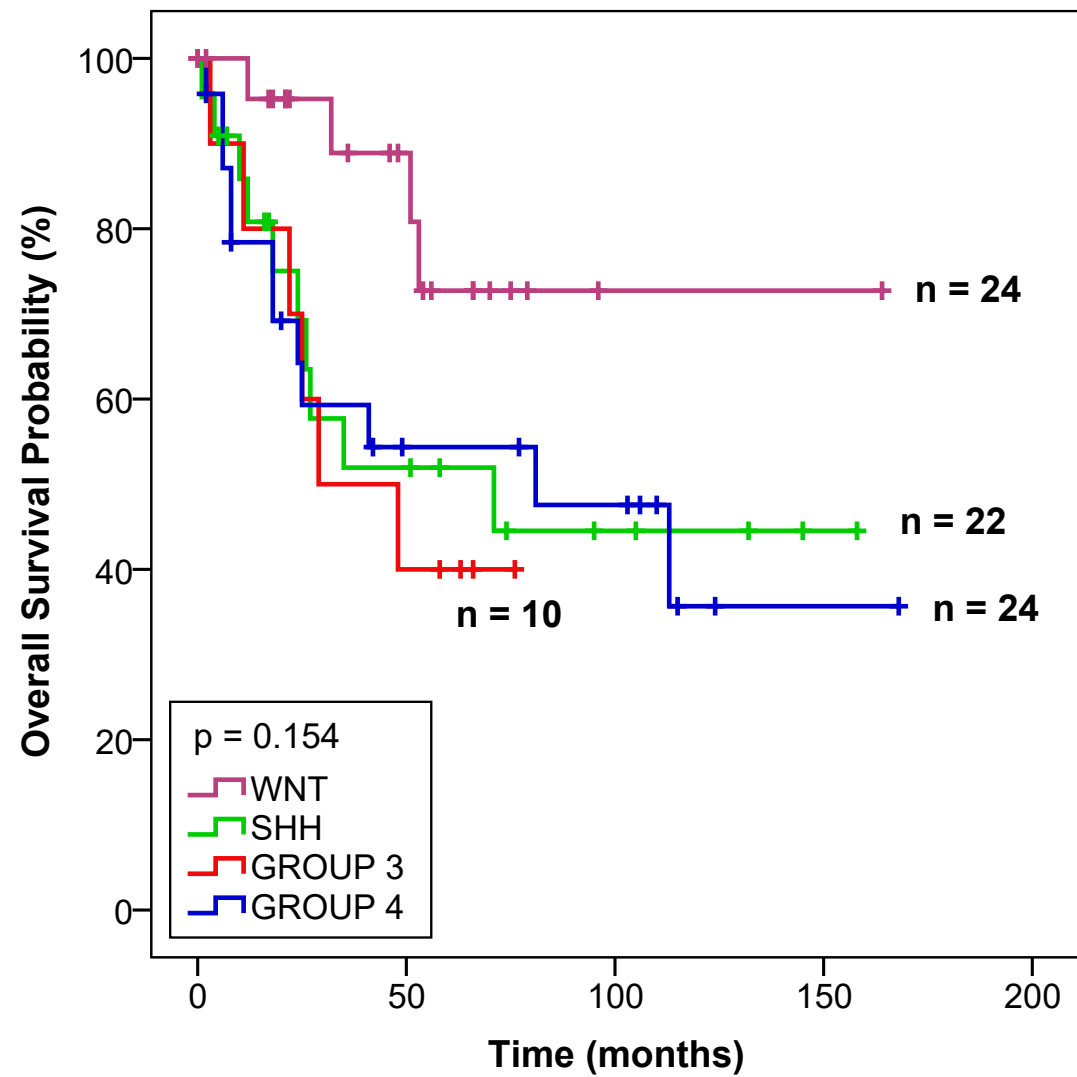

**Fig. S4** Overall survival of molecular subgroups ( n=80 )

Supplement: Supplementary file 7 — Figure S4. Overall survival of molecular subgroups (n = 80). (PDF 62 kb) [file 40478_2019_681_MOESM7_ESM.pdf]
